# The Generation of Dual-Targeting Fusion Protein PD-L1/CD47 for the Inhibition of Triple-Negative Breast Cancer

**DOI:** 10.3390/biomedicines10081843

**Published:** 2022-07-30

**Authors:** Yanlin Bian, Tong Lin, Tanja Jakos, Xiaodong Xiao, Jianwei Zhu

**Affiliations:** 1 Engineering Research Center of Cell & Therapeutic Antibody, Ministry of Education, School of Pharmacy, Shanghai Jiao Tong University, Shanghai 200240, China; weixiaobyl@sjtu.edu.cn (Y.B.); lintong0802@163.com (T.L.); tanja.jakos@sjtu.edu.cn (T.J.); 2 Jecho Biopharmaceuticals Co., Ltd., No. 2018 Zhongtian Avenue, Binhai New Area, Tianjin 300457, China; xiaodong.xiao@jecholabs.com; 3 Jecho Laboratories Inc., 7320 Executive Way, Frederick, MD 21704, USA

**Keywords:** dual-targeting protein, PD-L1, CD47, TNBC, tumor inhibition

## Abstract

Triple-negative breast cancer (TNBC) is a highly aggressive subset of breast cancer with limited therapeutic options. However, its immune evasion mechanisms, characterized by the over-expression of the immune checkpoint molecules PD-L1 and CD47, can be targeted in order to facilitate cancer elimination by cells of innate and adaptive immunity. In this paper, we describe the design, preparation, and evaluation of three novel dual-targeting fusion proteins that were based on the structure frame of prototype IAB (innate and adaptive dependent bispecific fusion protein) and the “Orcutt-type IgG-scFv” molecular model. Three molecules with different spatial conformations were designed to improve antigen–antibody affinity by the addition of Ag–Ab binding sites from the variable region sequences of the anti-PD-L1 monoclonal antibody (mAb) atezolizumab and CV1, a high-affinity receptor of CD47. The results showed that the best-performing among the three proteins designed in this study was protein Pro3; its CV1 N-terminus and Fc domain C-terminus were not sterically hindered. Pro3 was better at boosting T cell proliferation and the engulfment of macrophages than the IAB prototype and, at the same time, retained a level of ADCC activity similar to that of IAB. Through improved design, the novel constructed dual-targeting immunomodulatory protein Pro3 was superior at activating the anti-tumor immune response and has thus shown potential for use in clinical applications.

## 1. Introduction

Triple-negative breast cancer (TNBC) is a subtype of breast cancer (BC) that lacks the immunohistochemical expression of the estrogen receptor (ER), the progesterone receptor (PR), and the human epidermal growth factor receptor-2 (HER-2). TNBC has been characterized as highly aggressive and hard to treat; it has a poor prognosis [1] and represents approximately 10–19% of all breast cancer cases [2]. Distinct subpopulations of immune cells are known to have specific impacts on the function of the anti-tumor immune response. According to published reports [3,4,5], TNBC is associated with elevated intratumoral levels of both tumor-infiltrating lymphocytes (TILs) and tumor-associated macrophages (TAMs), along with a relatively high degree of expression of various immune checkpoints, such as programmed death ligand-1 (PD-L1) and CD47. Therefore, the need to restore the balance in the TNBC micro-environment provides a strong rationale for immunotherapies, especially for the use of the immune checkpoint blockade method.

Native regulatory mechanisms, including immune checkpoint pathways, have been investigated to prevent collateral damage from immune cells’ unrestrained activation. However, these same pathways can be exploited by tumors during immune evasion [6]. PD-L1 is an inhibitory ligand that is over-expressed by many human tumors. It can induce a negative signal of “do not find me” through engagement with its programmed death-1 receptor (PD-1) on the T lymphocyte surface, leading to reduced T cell proliferation, cytokine production, and cytotoxic functions [7,8]. Similarly, the increased expression of CD47 is involved in another tumor-evasion mechanism, in which interaction with its receptor signal regulatory protein-α (SIRP-α) triggers an inhibitory “do not eat me” signal for phagocytic immune cells [9]. An increased understanding of the role of the immune system in tumor progression has revealed critical mechanisms by which TNBC escapes both innate and adaptive immunity; this knowledge provides the opportunity to target these inhibitory checkpoints.

Recent work has demonstrated the great potential of synergistic anti-tumor effects through the dual-blocking of the PD-1/PD-L1 and CD47/SIRP-α immune checkpoint pathways. For instance, avelumab, a fully human IgG1 anti–PD-L1 mAb, was shown to increase the secretion of interferon γ (IFNγ) from anti-tumor immune effector cells, which could enhance both PD-L1 expression and antibody-dependent cellular cytotoxicity (ADCC)-mediated lysis [10]. In addition, macrophages activated by the anti-CD47 antibody were able to initiate better T cell responses through enhanced CD8+ T cell cross-priming [11]. Furthermore, Liu’s [9] CD47/PD-L1 dual-targeting fusion protein (denoted as IAB) illustrated its synergistic functionality by activating both innate and adaptive immune responses in vivo. However, IAB had lower antigen–antibody affinity and bioactivity in vitro than its parental clones, mostly due to its single antigen–antibody (Ag–Ab) binding arm. This suggested that the enhancement of anti-TNBC cytotoxicity might be achieved through structural reconstruction by the addition of Ag–Ab binding sites.

A novel bispecific antibody format reconstructed by Orcutt et al. [12] was generated as a normal IgG1-like mAb; its light chains were fused, at the C-terminus, with scFv or peptides that recognized another antigen. This molecular model of an “Orcutt-type IgG-scFv” showed increased antigen affinity, good serum half-life, and an adequately stabilized structure [13].

**Figure 1:**
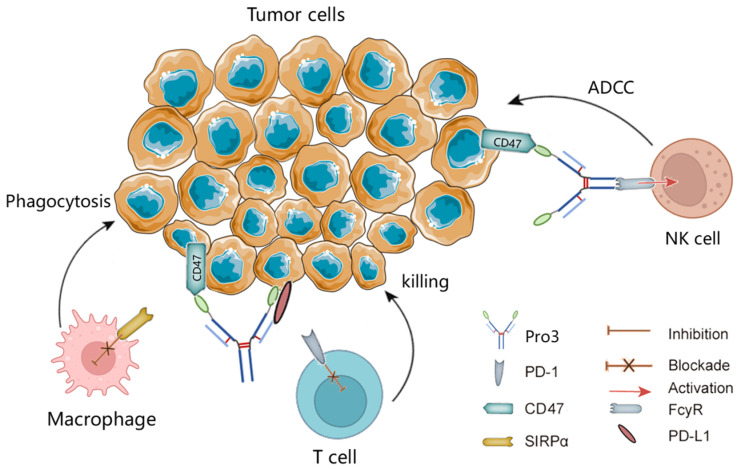
Schematic illustration of blockades of PD-L1 and CD47 by the dual-targeting fusion protein Pro3. ADCC, antibody-dependent cell-mediated cytotoxicity; NK, natural killer; SIRPα, signal-regulatory protein α.

Therefore, based on the specific immune signature of the TNBC tumor environment, where the immune checkpoint molecules CD47 and PD-L1 are over-expressed, we present here three novel dual-targeting fusion proteins for the treatment of TNBC. A schematic overview of the activity of the dual-targeting fusion protein in cancer is shown in Figure 1. Based on the structure frame of prototype IAB and the “Orcutt-type IgG-scFv” molecular model, we designed three novel proteins with different spatial conformations (denoted as Pro1, Pro2, and Pro3). Our improved design includes Ag–Ab binding sites from the variable region sequences of the anti-PD-L1 mAb atezolizumab [14] and from CD47’s high-affinity receptor CV1 [15]. Further, we performed a series of biochemical and biological activity assays to evaluate target binding affinities, tumor-directed cytotoxicity, ADCC, the stimulation of IFN-γ secretion, and phagocytosis. This study was facilitated by the significant experience of the authors with protein–protein interactions [16], bispecific antibody platforms [17,18,19], and T-cell-engaging dual function anti-tumor molecules [20,21].

## 2. Materials and Methods

### 2.1. Strains, Cell Lines, and Other Reagents

The competent strain of *E. coli* DH5α was produced in-house. The plasmids containing light and heavy chains of IgG1 Ab (pM09/CD3-L2 and pM09/5D9 Hc), variable regions of anti-human PD-L1 mAb atezolizumab (pET22b/PD-L1 scFv, pM09/PD-L1-Hc, and PM09/PD-L1-Lc), anti-PD-L1 scFv with cysteine mutation (pET22b/PD-L1 scFv*), CD47-affinity receptor CV1 (pET22b/CV1, pM09/CV1-Fc, and pM09/Fc-CV1), and variable regions of anti-human CD47 mAb B6H12 (pUC57/B6H12-Lc and pUC57/B6H12-Hc) were all prepared from constructs stored at the Engineering Research Center of Cell & Therapeutic Antibody of MOE, China, at Shanghai Jiao Tong University (Shanghai, China).

The mammalian cell lines HEK293E and MDA-MB-231 (TNBC) were obtained from the American Type Culture Collection (ATCC, Rockville, MD, USA). The cell line MCF-10A (non-TNBC) was kindly gifted by Prof. Han Xianghui’s group at Longhua Hospital (Shanghai, China). Human peripheral blood mononuclear cells (PBMCs) were obtained from the venous blood of healthy donors. Human macrophages, dendritic cells (DC), and CD4^+^ T lymphocytes were all induced or isolated from fresh PBMCs.

The positive control antibodies anti-PD-L1 mAb (atezolizumab) and anti-CD47 mAb (B6H12) were produced in-house from previously stored plasmids: pM09/PD-L1-Hc and pM09/PD-L1-Lc, and pUC57/B6H12-Lc and pUC57/B6H12-Hc, respectively. The structure controls CV1-Fc and Fc-CV1 were obtained from the plasmids pM09/CV1-Fc and pM09/Fc-CV1, respectively. In addition, the prototype IAB was prepared according to the procedure outlined in published work [9].

### 2.2. Molecular Design and General Construction of Dual-Targeting Proteins

According to the structure frame of prototype IAB and the “Orcutt-type IgG-scFv” molecular model, three novel dual-targeting proteins of diverse spatial conformations were developed. They all contained symmetrical Ag–Ab binding sites with the variable region sequences of anti-PD-L1 mAb atezolizumab [14] and CD47’s [15] high-affinity receptor CV1. The information about DNA sequences is provided in Appendix A.

Consistent with Orcutt’s structure design, the CV1 was fused to the C-terminus of the light chain of the anti-PD-L1 IgG to create the novel protein Pro1, in which an intra-molecular interchain disulfide bond (through cysteine mutation at VH G44C and VL Q100C) was introduced for higher molecular stability [12,13]. The heavy chain was the same as that of human IgG1, and the light chain was constructed as leader-VL*-Cκ-(G_4_S)2-CV1, wherein the VL* was a variable light domain of atezolizumab, with a site mutation at Q100C. The novel proteins Pro2 and Pro3 were subsequently constructed by the fusion of CV1 at the C-terminus or N-terminus of anti-PD-L1 IgG; the heavy chain was VH-CH1-Fc-(G4S)4-CV1 (Pro2) or CV1-(G4S)2-VH-CH1-Fc (Pro3), and the light chain was the same as that of normal IgG1 for both proteins. All the fragments mentioned above were cloned into a separate pM09-vector. According to established protocols [12], the plasmids were transiently co-transfected into HEK293E cells by the polyethyleneimine (PEI) method [22] at the optimized weight ratios (Hc:Lc) of 1:1, 1:2, and 1:2, respectively, for Pro1, Pro2, and Pro3. The novel proteins Pro1, Pro2, and Pro3 were subsequently expressed and purified by MabSelect SURE chromatography (GE Healthcare, Shanghai, China) following the manufacturer’s instructions. Detailed information about the construction of the novel dual-targeting proteins is provided in the Supplementary Materials (Figure S1 and Tables S1–S4).

### 2.3. Binding Analysis In Vitro

The dual-antigen binding affinities of the three novel proteins were evaluated by bio-layer interferometry (BLI), which was conducted on a PALL ForteBio Octet RED96 system. The total working volume for the samples or buffers was 0.2 mL per well, and the working temperature was set at 37 °C. Briefly, streptavidin-coated biosensor tips were pre-wetted in phosphate-buffered saline (PBS), followed by the loading of the biotin-conjugated antigens PD-L1 or CD47 (100 nM, Sino Biological Inc., Beijing, China). Afterwards, a series of protein samples, including the positive controls (anti-PD-L1 mAb and anti-CD47 mAb), the prototype (IAB), and the three novel proteins (Pro1, Pro2, and Pro3), were associated with the ligands in concentrations of 25 nM, 50 nM, 75 nM, 100 nM, 125 nM, and 150 nM. Finally, the dissociation step was conducted by dipping the sensors in PBS. Analysis was performed with Octet software, during which the association and dissociation signals were baseline corrected, and global fit was used to calculate the affinity and rate constants. Accordingly, the association rate constant (k_a_) indicated the Ag–Ab complex formation rate per second in a 1 M solution, and the dissociation rate constant (kd) defined the stability of the Ag–Ab complex. The affinity constant K_D_ was calculated by the formula k_d_/k_a_ [23,24].

### 2.4. PD-L1 and CD47 Co-Expression on MDA-MB-231 Cells

The co-expression of the antigens PD-L1 and CD47 on the TNBC cell line MDA-MB-231 was analyzed by flow cytometry. Tumor cells (5 × 10^5^) were incubated with 1 μg of anti-PD-L1 mAb or anti-CD47 mAb (both made in-house). FITC-conjugated anti-human Fc IgG (Jackson ImmunoResearch, West Grove, PA, USA) was used as the secondary antibody. Samples were measured on a CytoFLEX system (Beckman Coulter, Shanghai, China), and results were analyzed by CytExpert software (Beckman Coulter, Sahnghai, China).

### 2.5. In Vitro Human Macrophage Activation

M1-type macrophages were induced in vitro from freshly isolated human PBMCs, with an initial treatment of M-CSF (Sino Biological Inc., Beijing, China) at a concentration of 25 ng/mL for 6 days and a subsequent exchange to a buffer containing a combination of M-CSF (25 ng/mL) plus IFN-γ (50 ng/mL, Sino Biological Inc.) for an additional 24 h. Cells at 1 × 10^5^ cells/well of mature macrophages were co-plated with 2 × 10^5^ MDA-MB-231 cells. The cells were labeled with 1 μM carboxyfluorescein diacetate succinimdyl ester (CFSE, Invitrogen, Wuhan, China) that was diluted in a serum-free RPMI 1640 medium (Gibco, Waltham, MA, USA) in a 48-well plate (Corning, Corning, NY, USA). Afterwards, 100 nM of antibodies (anti-CD47 mAb, CV1-Fc, Fc-CV1, IAB, Pro1, Pro2, and Pro3) or an IgG1 isotype control (Sino Biological Inc.) was added and incubated for 4 h at 37 °C. Next, the cells were washed in PBS and gently resuspended in TrypLE (Gibco, Waltham, MA, USA), followed by staining with 1 μg of APC-conjugated anti-CD11b antibody (Bioscience). After the final washing step, all the samples were analyzed on a CytoFLEX system (Beckman Coulter), and the phagocytic index was calculated by GraphPad Prism 8 in accordance with the ratio of CFSE+ macrophages, as previously described [25].

### 2.6. Mixed Lymphocyte Reaction (MLR)

CD4^+^ T cells were isolated from fresh PBMCs using a magnetic-bead cell separation kit (STEMCELL Technologies, Shanghai, China). DC cells were prepared by culturing PBMCs in vitro for 6 days with human IL-4 (20 ng/mL, Sino Biological Inc.) and GM-CSF (50 ng/mL, Peprotech, Cranbury, NJ, USA), followed by 24 h stimulation with lipopolysaccharides (LPS, 1 μg/mL). CD4^+^ T cells (1 × 10^5^) and mature DC cells (1 × 10^4^) were co-cultured with control proteins (anti-PD-L1 mAb, CV1-Fc, Fc-CV1, and IAB) or the proteins Pro1, Pro2, and Pro3, with three concentrations for all three testing groups: 2.4 nM, 12 nM, and 60 nM. After 5 days, the secretion of cytokine IFN-γ in cell culture supernatants was analyzed by ELISA (R&D systems, Minneapolis, MN, USA) as per the manufacturer’s instructions.

### 2.7. Antibody-Dependent Cell-Mediated Cytotoxicity (ADCC)

The ADCC effect was evaluated using a lactate dehydrogenase (LDH) measurement kit (Promega, Durham, NC, USA), according to published work [26]. MDA-MB-231 cells expressing both PD-L1 and CD47 were seeded at a concentration of 5000 cells/100 μL in a 96-well plate. After 20 h, serially diluted controls (anti-PD-L1 mAb, CV1-Fc, Fc-CV1, and IAB) or the proteins (Pro1, Pro2, and Pro3), together with 2 × 10^5^ PBMCs per well, were added to the target cells and incubated for another 15 h. Afterwards, the LDH kit was utilized for the quantification of cytotoxicity mediated by antibodies in a colorimetric assay, where the measured absorbance was proportional to the fraction of lysed cells.

## 3. Results

### 3.1. Molecular Construction and Preparation of the Dual-Targeting Proteins

**Figure 2:**
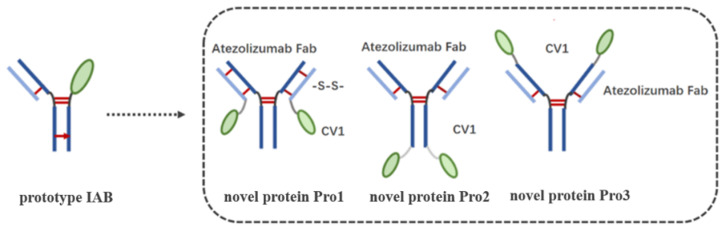
Design and schematic representation of the structures of the three dual-targeting proteins. The left “prototype IAB” is the structure of prototype IAB [12]. Prototype IAB was developed using “knobs into holes” technology, which is represented by a red arrow.

TNBC is a highly aggressive cancer with limited therapeutic options. However, it is characterized by high contents of both TILs and TAMs, along with over-expressed immune checkpoints, including PD-L1 and CD47; these characteristics suggest a strong rationale for immune checkpoint blockade therapy [3,4,5]. Recently, the anti-CD47/PD-L1 dual-targeting fusion protein IAB showed great potential for improving host immune responses in vivo [9], although it had sub-optimal antigen binding affinity and reduced biological activity in vitro on account of its “single-arm binding site”. In this study, based on the available information regarding the tumor-immune microenvironment in TNBC and the over-expression of the well-characterized immune checkpoints PD-L1 and CD47, we reconstructed the molecular frame of prototype IAB. Our aim was to enhance bioactivity through the addition of Ag–Ab binding sites, with reference to Orcutt’s study [12], in which a novel format of bispecific Ab (bsAb) managed to retain both parental affinities and had good stability and in vivo half-life. Thereby, three novel dual-targeting proteins (Pro1, Pro2, and Pro3) that contained the variable region sequences of atezolizumab [14] and the CV1 monomer [15] were designed and developed; CV1 was fused to anti-PD-L1 IgG’s Lc C-terminus and to the Hc C-terminus or N-terminus, respectively, as shown in Figure 2.

**Figure 3:**
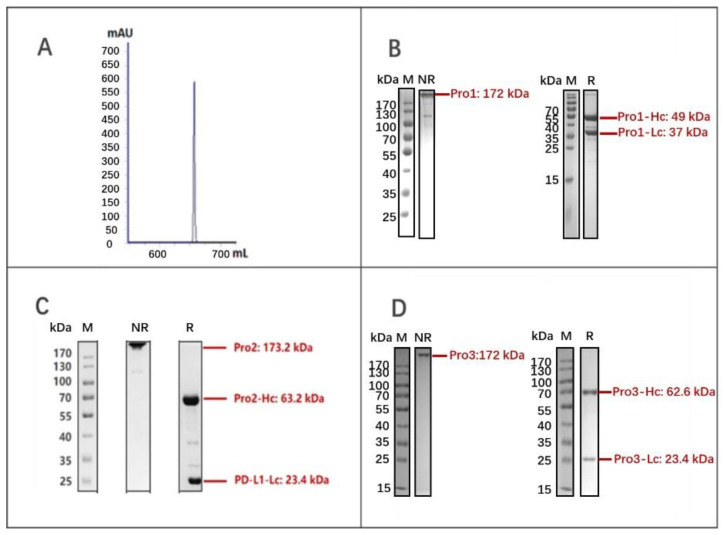
Expression and purification of dual-targeting fusion proteins. (**A**) Elution curve of Pro1 after MabSelect SURE purification; (**B**) SDS-PAGE analysis of Pro1 after affinity purification; (**C**) SDS-PAGE analysis of Pro2; (**D**) SDS-PAGE analysis of Pro3. (M: marker; NR: non-reduced samples; R: reduced samples).

After transient expression by the PEI-mediated transfection of HEK293E cells, the three dual-targeting proteins were purified by MabSelect SURE chromatography and analyzed by SDS-PAGE under non-reducing and reducing conditions. As shown in Figure 3A,B, the molecular weight (MW) of Pro1 was in good agreement with the expected value of around 172 kD for a fully assembled product; it also appeared to exhibit the desired purity. The yield of Pro1 was calculated as 43.4 mg/L by absorbance measurement, using the following formula: OD_280_/extinction coefficient [27]. Likewise, Pro2 (173.2 kD) and Pro3 (172 kD) were both correctly assembled as full-length proteins with relatively high purity, yielding 26.5 mg/L and 17.3 mg/L, respectively. These results demonstrated that the novel dual-targeting proteins Pro1, Pro2, and Pro3 were successfully designed and prepared for further biological evaluations.

### 3.2. Binding Characterization In Vitro

**Figure 4:**
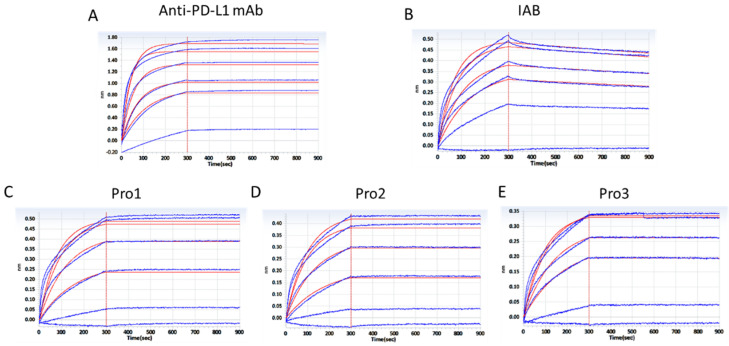
Affinity analysis of proteins binding to antigen PD–L1 through a BLI assay. (**A**) The positive control antibody; (**B**) prototype IAB; (**C**) Pro1; (**D**) Pro2; (**E**) Pro3. (Response units vs. time).

To confirm that the dual-antigen binding affinity of the reconstructed system was not disrupted by frame reconstruction, the affinities for PD-L1 and CD47 were measured by BLI assay using a ForteBio Octet RED96 system. The curves shown in Figure 4 are indicative of the antibodies’ typical association and disassociation rates for the antigen PD-L1. According to the K_D_ constants calculated in Table 1, it was deemed that all of the three reconstructed formats with additional Ag–Ab binding sites did retain the parental affinity to PD–L1. All three proteins showed a significant increase in binding affinity as compared to the prototype IAB, among which Pro1 demonstrated the highest affinity (<1.0 × 10^−12^).

**Figure 5:**
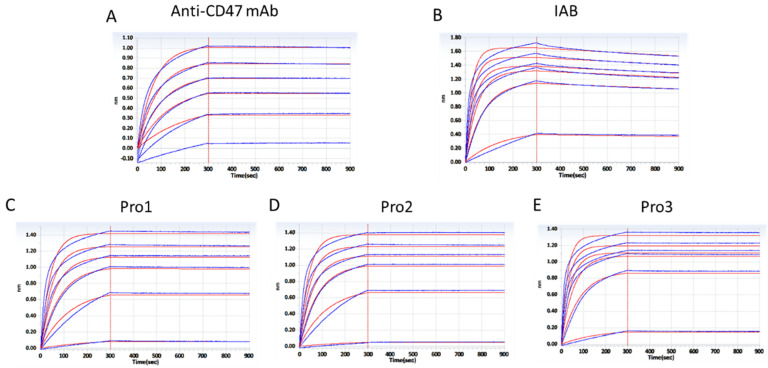
Affinity analysis of proteins to antigen CD47 through a BLI assay. (**A**) The positive control antibody; (**B**) prototype IAB; (**C**) Pro1; (**D**) Pro2; (**E**) Pro3. (Response units vs. time).

Accordingly, the association and disassociation rates of the controls and reconstructed proteins for the molecular target CD47 were measured, as presented in Figure 5. K_D_ constants, calculated in Table 2, showed that the three reconstructed formats exhibited similar affinities to CD47 compared to the parental antibody. The binding affinities of Pro1 (K_D_ = 9.138 × 10^−12^) and Pro3 (K_D_ = 9.918 × 10^−12^) were about 50-fold higher than that of IAB (K_D_ = 4.813 × 10^−10^), but there was no significant improvement seen with Pro2.

### 3.3. Co–Expression of PD–L1 and CD47 on MDA–MB–231

**Figure 6:**
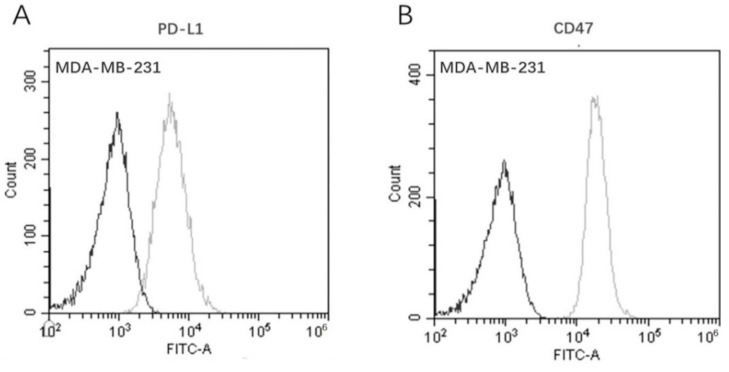
Co-expression of dual-targets on the TNBC cell line MDA-MB-231 by flow cytometry. (**A**) Antigen PD-L1; (**B**) antigen CD47. (Dark line: blank control; gray line: Ab-incubation group).

Even though clinical immuno-histochemical analysis showed that TNBC was densely populated with TILs and TAMs, the immune cells were not able to effectively eliminate the transformed tumor cells. This fact can be largely attributed to the high expression of the inhibitory [3,4] immune checkpoints PD-L1 and CD47 [5]. Therefore, we investigated the dual-antigen expression on MDA-MB-231 cells to confirm whether this TNBC tumor cell line was qualified to be used in further bioassays. As shown in Figure 6, FACS analysis confirmed that MDA-MB-231 cells express both PD-L1 and CD47, which was consistent with the clinical pathology features of TNBC [28].

### 3.4. In Vitro Human Macrophage Activation

**Figure 7:**
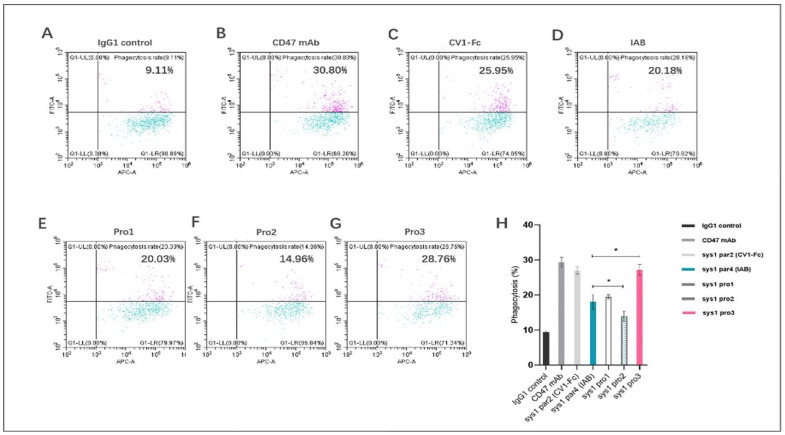
Novel reconstructed proteins induced human macrophages to phagocytize MDA-MB-231 tumor cells. The proportion of macrophages that engulfed tumor cells was measured by flow cytometry as a proportion of CFSE+CD11b+ cells. (**A**) Isotype IgG1; (**B**) positive control anti-CD47 mAb; (**C**) structure control CV1-Fc; (**D**) prototype IAI (**E**) Pro1; (**F**) Pro2; (**G**) Pro3. (**H**) The results are presented as mean ± standard deviation (SD), where * *p* < 0.05 is indicative of statistical difference.

To explore how well the dual-targeting proteins induced phagocytosis, we used CFSE-labeled human TNBC tumor cells MDA-MB-231 and matured macrophages in vitro. The ratio of CFSE+ macrophages was calculated after incubation with 100 nM of control (IgG1 isotype, anti-CD47 mAb, CF1-Fc, and the prototype IAB) or dual-targeting proteins (Pro1, Pro2, and Pro3). As shown in Figure 7A–D, the positive controls anti-CD47 mAb and CV1-Fc, as well as prototype IAB, were able to induce macrophage-mediated phagocytosis to a significant extent. The phagocytosis mediated by the dual-targeting proteins was dependent on their spatial conformations. As indicated in Figure 7G, the novel protein Pro3, in which the Fc domain was not hindered by CV1, exhibited significantly improved bioactivity. Pro3 induced phagocytosis of MDA-MB-231 cells in 27.25% ± 1.51% of macrophages, as opposed to 18.06% ± 2.13% measured for IAB protein (*p* < 0.05). Subsequently, Pro1, which had the Fc domain open but the N-terminus of CV1 blocked, performed similarly to IAB (Figure 7E); Pro2, in which both the Fc domain and N-terminus of CV1 were blocked, displayed much lower activity than the prototype (Figure 7F) (*p* < 0.05). The above results demonstrated that the three reconstructed proteins induced human macrophages to phagocytize MDA-MB-231 tumor cells at different levels due to differences in the spatial structure of the antigen–antibody binding interface and the constant region. The best-performing protein was Pro3; its CV1 N-terminus and Fc domain C-terminus were not sterically hindered (Figure 7H).

### 3.5. Dual-Targeting Proteins Exhibited Superior Activity in a T Cell Activation Assay

**Figure 8:**
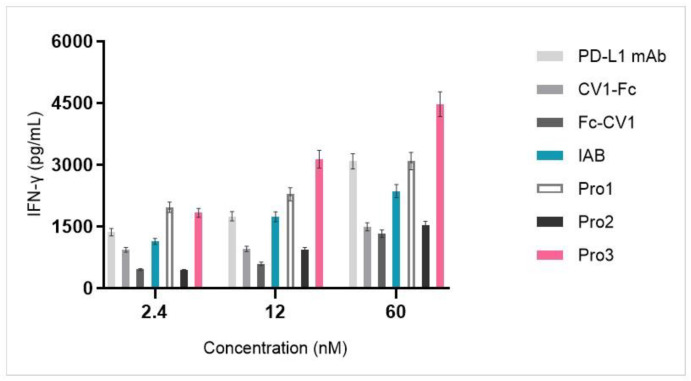
Dual-targeting proteins stimulated IFN-γ release from the MLR system in a dose-dependent manner.

The stimulation of T cells with the novel reconstructed proteins was evaluated in an allogeneic mixed lymphocyte reaction (MLR) system by using human CD4^+^ T cells and DC cells that were isolated from PBMCs. As shown in Figure 8, PD-L1 blockade by anti-PD-L1 mAb, IAB, and all of the three proteins enhanced IFN-γ release in a dose-dependent manner along a concentration range from 2.4 to 60 nM. The highest concentrations of IFN-γ were released by the novel Pro3, which had the C-terminus of anti-PD-L1 mAb open and CV1 attached at the heavy chain N-terminus. A quantity of 60 nM of Pro3 stimulated the release of over 4000 pg/mL of inflammatory cytokine IFN-γ. Owing to the exposed N-terminus of anti-PD-L1 mAb, Pro1 also induced a higher concentration of IFN-γ than prototype IAB; however, Pro2, which had an open binding domain towards PD-L1 but blocked CV1, had lower activity when compared with IAB.

### 3.6. Dual-Targeting Proteins Retained the ADCC-Mediated Capacity of IAB

Although most of the fully human or humanized antibodies targeting PD-1 or PD-L1 are of the IgG4 isotype or mutated IgG1 Fc domain with low levels of ADCC activity [29], the anti-PD-L1 mAb avelumab can induce ADCC. Nevertheless, it was proved that avelumab administration was safe and did not trigger the lysis of PD-L1+ immune cells [30]. Furthermore, research confirmed that FcγRs’ engagement could enhance the tumor inhibitory capability of the anti-PD-L1 antibody. Therefore, a combination of the normal IgG1 Fc domain and anti-PD-L1 mAb offers potential anti-tumor resistance. Here, the ability of the novel proteins to mediate ADCC activity in vitro was tested in an LDH assay.

**Figure 9:**
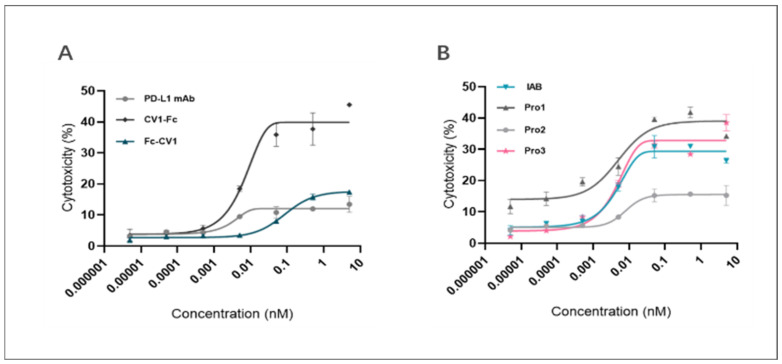
Reconstructed proteins and control proteins mediated ADCC against MDA-MB-231 cells. The plots show a dose-dependent increase in the proportion of lysed tumor cells for (**A**) controls (anti-PD-L1 mAb, CV1-Fc, Fc-CV1) and (**B**) reconstructed proteins (Pro1, Pro2, and Pro3).

As Figure 9A indicates, CV1-Fc had higher ADCC activity than anti-PD-L1 mAb, suggesting the former molecule can augment cytotoxicity towards MDA-MB-231 cells. Meanwhile, Fc-CV1, with the C-terminus of the heavy chain blocked, exhibited much lower cytotoxicity in comparison to CV1-Fc, for which the corresponding domain was kept free and open (17.59% vs. 42.95% for top cytotoxicity). The results further underline the importance of the correct spatial orientation of each part in the multi-domain assembly.

The results of the ADCC activity test for the proteins are presented in Figure 9B and Table 3. In the concentration range from 10^−4^ nM to 10^−1^ nM, IAB, Pro1, and Pro3 all displayed significant ADCC-activating capability. When compared with the prototype, Pro1 showed relatively similar ADCC effects to Pro 3 on MDA-MB-231 cells (23.57% vs. 25.04% for the cytotoxicity span), while Pro2, at the same concentration gradient, showed a significant loss of activity (~5% to 29.36% vs. ~5% to 15.54% for the span). On the other hand, the ADCC effects of Pro3 were successfully retained, as the cytotoxicity span was 1.47 ~ 35.71%. These results suggest that the spatial format of Pro2 had a critical impact on Fc-mediated immune responses that was even more pronounced than that of the prototype. This illustrates, once again, that careful consideration of different spatial orientations is needed in order to achieve the desired functionality of reconstructed molecules.

## 4. Discussion

TNBC has been characterized as highly aggressive, and it is harder to treat compared to other breast cancer subtypes [4]. However, the significant infiltration of both TILs and TAMs, along with the over-expression of immune checkpoints including PD-L1 and CD47, has provided a strong rationale for immune checkpoint blockade therapy [3,4,5]. In addition, published work [8] indicated that an anti-CD47/PD-L1 dual-targeting fusion protein IAB could restore the host’s immune response in vivo by activating both innate and adaptive immunity for more effective tumor eradication. Nevertheless, the IAB could be improved further, as it has sub-optimal antigen-binding affinity and relevant bio-functions in vitro because of its “single-arm binding site”. In order to enhance the bioactivity of the dual-targeting PD-L1/CD47 protein, we reconstructed the molecular frame of prototype IAB. The novel format provided additional Ag–Ab binding sites and, at the same time, exhibited sufficient molecular stability, as shown in another study by Orcutt et al. [12]. This format of bsAb managed to retain parental antibody affinities and had good stability or clearance in vivo [12]. Therefore, three novel dual-targeting proteins (denoted as Pro1, Pro2, and Pro3) that utilize the variable region sequences of atezolizumab [14] and the CV1 monomer [15] were developed, with a CV1-fusion at the anti-PD-L1 IgG’s Lc C-terminus and the Hc C-terminus or N-terminus, respectively. Their multi-biological effects on the TNBC cell line MDA-MB-231 were studied to highlight the structure–activity relationship and provide preclinical data that would support their further development.

The data from our study indicated that dual-targeting proteins with additional Ag–Ab binding sites could mediate diverse levels of anti-TNBC activity, dependent on their different spatial conformations.

Published work highlighted the importance of specific sites at the N-terminus of the CV1 monomer for binding to its target CD47 [31]. The fusion of even short peptides at this site could significantly impact CV1’s affinity and bioactivity [31]. Therefore, it was expected that the optimal affinity of our reconstructed proteins, which not only offer the benefits of CD47/SIRPα blockade but also of Fc-mediated phagocytosis, would require an unobstructed N-terminus of CV1. Simultaneously, the function of the Fc domain is critical for various stimulatory immune pathways (e.g., Fc/FcγR I for mediating phagocytosis) [32]. Consistent with the evidence above, Pro3, with the CV1 N-terminus and the functional Fc domain exposed, exhibited significantly enhanced bioactivity, in terms of inducing the phagocytosis of MDA-MB-231 cells, in comparison to the prototype.

According to Wang’s study [33], the expression of molecular PD-1 and PD-L1 were both upregulated in an allogeneic MLR system composed of CD4^+^ T cells and DC cells. The relevant inhibitors of this pathway could enhance the release of pro-inflammatory cytokine. As we explored the influence of the novel proteins on T cell activity, we determined that all of the three novel proteins stimulated IFN-γ release in a dose-dependent manner. Among them, Pro3, with an open C-terminus of anti-PD-L1 mAb and an open N-terminus of CV1, had the highest level of activity, with the highest concentrations of IFN-γ released, reaching over 4000 pg/mL. Further, research by Soto-Pantoja [34] and Cui Lei [35] confirmed that CD47 was over-expressed on activated T lymphocytes and that they contributed to another inhibitory immune pathway of CD47/thrombospondin-1 (TSP-1). Its blockade by an anti-CD47 targeting drug also promoted the activation and proliferation of T cells. Therefore, along with the published evidence, our assumption about the disrupted spatial conformation of Pro2’s CV1 monomer could explain its relatively low level of activity. The molecular conformation of IgG-like targeting antibodies was highly linked to their biological functions, such as ADCP; however, the situation was different, for example, for ADCC. It is believed that ADCC is mediated mainly through the Fc domain of CH2/CH3 binding to Fc/FcγRIII expressed on NK cells. Orcutt-type proteins (the prototype of Pro1) have been reported to retain serological half-lives that are close to that of IgG mAbs through FcRn binding with the Fc domain of CH2/CH3 [36].

By performing an ADCC activity assay in vitro, we observed that CV1-Fc showed higher activity than anti-PD-L1 mAb. This is in accordance with the results evaluating the co-expression of PD-L1 and CD47 on MDA-MB-231 cells, which showed a higher MFI for antigen CD47, suggesting that CD47 might dominate in a cytotoxicity model with MDA-MB-231 cells. The ratio of effector cells to target cells might explain the relatively narrow cytotoxicity span of the anti-PD-L1 mAb [37]. Furthermore, as ADCC activity depends on Ag–Ab binding affinity [37] along with the spatial constraints of functional Fc [32], the reconstructed format of Pro3 with an open CV1 N-terminus and an open Fc domain had the best bioactivity in this assay as well.

Our results comprehensively showed that, for leveraging the immune system to treat TNBC, the novel reconstructed proteins displayed markedly improved biological activity. They were designed by combining the structure frame of prototype IAB and the “Orcutt-type IgG-scFv” molecular model, with different spatial conformations taken into account. Among the proteins tested, Pro3 enhanced the engulfment of macrophages and T cell activation and, at the same time, retained a similar level of ADCC as the original molecule IAB. Herein, we describe the development of a novel candidate for an anti-cancer drug, namely Pro3, which offers improved inhibitory potential against TNBC cancer cells.

Recently, a variety of studies confirmed the synergistic anti-tumor effects of the simultaneous activation of both innate and adaptive immunity through multiple cross-priming mechanisms. For instance, the therapeutic efficacy of the anti-HER2/neu antibody [38] and of the anti-EGFR mAb cetuximab [39] partially depends on both natural killer cells and T cells. Moreover, atezolizumab, in combination with nab-paclitaxel, extended the progression-free survival (PFS) of TNBC patients [38], providing a strong rationale for exploring new therapeutic combinations for controlling tumors that are resistant to first-generation antibodies.

A study indicated that the dual blockade of CD47 and PD-L1 overcomes innate and adaptive immune resistance to antibody immunotherapy and substantially enhances anti-tumor responses [40]. The combination of anti-CD47 and PD-1/PD-L1 has been studied by different research groups [41]. Currently, multiple clinical trials are in phase I or phase II, such as one using a combination of ALX148 and pembrolizumab in head and neck squamous cell carcinoma (NCT03013218) [42], one using IMM01 and tislelizumab in advanced solid tumors (CTR20220791), and one using Hu5F9-G4 with different immunotherapies [43]. Among them, Hu5F9-G4 (5F9, magrolimab) is a first-in-class monoclonal antibody that blocks CD47. In terms of treatment, 5F9 is being tested in different treatment schemes in combination with different immunotherapies targeting PD-1/PD-L1, including avelumab (NCT03558139) [44], pembrolizumab (NCT04788043 and NCT04854499) [45], and atezolizumab (NCT03922477 and terminated). More clinical research is needed to adequately confirm the security and efficacy of these combinations in clinical practice.

Co-targeting PD-1/PD-L1 and CD47 with mAb combinations showed increased anti-tumor responses in clinical studies. However, CD47 mAbs are hindered by ubiquitous CD47 expression, leading to rapid target-mediated clearance and safety concerns. Consequently, dual-targeting CD47xPD-L1 bsAbs, enabling the preferential inhibition of CD47 on PD-L1-positive cells, are being tested as an alternative approach [46]. There are currently a number of bispecific antibodies targeting CD47 and PD-1/PD-L1 for the treatment of patients with various kinds of cancers [47], such as HX009 (NCT04886271), IBI322 (NCT04338659), PF-07257876 (NCT04881045) [48], SG12473 (CTR20211029), etc. Among them, the anti-PD-1/CD47 bsAb HX009 developed by Hangzhou Hanx Biopharmaceutics, Inc. to treat patients with advanced solid tumors, including gastric cancer, colorectal cancer, and liver cancer, has shown promising clinical data. The anti-tumor activities of this approach and the objective responses in multiple tumor types [49], along with the role of bsAb, are now undergoing further investigations in a phase Ib/II study (NCT04886271). Another candidate, named IBI322, is a recombinant anti-human CD47/PD-L1 bsAb developed by Innovent Biologics Co. Ltd.(Suzhou, China); it has demonstrated promising efficacy signals and a favorable safety and tolerability profile [50]. Several phase Ib trials have been conducted to further explore the safety and efficacy of IBI322 in multiple indications (NCT04338659, NCT04795128, and NCT04912466).

A non-clinical study indicated that CD47 targeted monotherapy, or a combination with anti-PD-L1, preserves T cell bioenergetics and anti-tumor function, resulting in a decreased TNBC tumor burden [51]. However, there are few relevant clinical trials of the above dual-targeting combination as applied to TNBC, so future research should consider the verification and exploration of the molecular and cellular mechanisms of the dual-blockade of the immune checkpoint pathways PD-1/PD-L1 and CD47/SIRPα by our novel reconstructed protein Pro3.

## 5. Conclusions

This work successfully achieved the generation of dual-targeting fusion proteins with anti-PD-L1/CD47 functions. We described the design, preparation, and evaluation of three novel dual-targeting fusion proteins (denoted as Pro1, Pro2, and Pro3) that were based on the structure frame of prototype IAB and the “Orcutt-type IgG-scFv” molecular model. The three molecules with different spatial conformations were designed to improve antigen–antibody affinity by the addition of Ag–Ab binding sites from the variable region sequences of anti-PD-L1 mAb atezolizumab and CV1, a high-affinity receptor of CD47. According to the in vitro analysis by biolayer interferometry, the novel reconstructed proteins with increased antigen binding sites all retained the molecular binding affinities targeting CD47 and PD-L1 and showed a significant improvement over prototype IAB. The multi-biological effects towards the TNBC cell line MDA-MB-231 demonstrated different levels of biofunctional mediation due to differences among the three constructs in the spatial structure of the antigen–antibody binding interface and the constant region. Pro3 was better at boosting T cell proliferation and the engulfment of macrophages than IAB prototype, and it retained a level of ADCC activity similar to that of IAB. In summary, the novel dual-targeting fusion protein Pro3 demonstrated stronger TNBC cancer cell inhibitory activity and has potential for clinical applications. Meanwhile, our findings provide a research basis for the structural modification and development of anti-tumor pharmaceuticals targeting multiple immune checkpoints.

## Figures and Tables

**Table 1:** Rate and equilibrium constants of proteins binding to PD–L1.

Antibody	K_a_ 1/Ms	K_d_ 1/s	K_D_ M	R^2−^
Anti-PD-L1 mAb	1.82 × 10^5^	4.48 × 10^−6^	2.469 × 10^−11^	0.9834
IAB	9.50 × 10^4^	1.80 × 10^−4^	1.894 × 10^−9^	0.9786
Pro1	8.40 × 10^4^	1.0 × 10^−7^	<1.0 × 10^−12^	0.9683
Pro2	7.52 × 10^4^	2.85 × 10^−7^	3.794 × 10^−12^	0.9861
Pro3	7.21 × 10^4^	3.05 × 10^−7^	4.229 × 10^−12^	0.9863

**Table 2:** Rate and equilibrium constants of antibodies binding to CD47.

Antibody	K_a_ 1/Ms	K_d_ 1/s	K_D_ M	R^2^
Anti-CD47 mAb	9.79 × 10^4^	3.99 × 10^−7^	4.076 × 10^−12^	0.9930
IAB	2.57 × 10^5^	1.24 × 10^−4^	4.813 × 10^−10^	0.9937
Pro1	1.60 × 10^5^	1.46 × 10^−6^	9.138 × 10^−12^	0.9954
Pro2	1.65 × 10^5^	1.98 × 10^−6^	1.202 × 10^−11^	0.9954
Pro3	2.42 × 10^5^	2.40 × 10^−6^	9.918 × 10^−12^	0.9919

**Table 3:** Dual-targeting proteins retained ADCC cytotoxicity towards MDA-MB-231 cells (E:T = 40:1).

Antibody	EC_50_ (ng/mL)	Span (%)	R^2^
IAB	0.56	23.57	0.9666
Pro1	0.86	25.04	0.9194
Pro2	1.35	10.50	0.9468
Pro3	0.76	34.24	0.9599

## Data Availability

The dataset supporting the conclusions of this article is included within the article.

## Supplementary Materials

The following supporting information can be downloaded at: https://www.mdpi.com/article/10.3390/biomedicines10081843/s1, Figure S1: Molecular schematic and plasmid constructions of three dual-targeting proteins; Table S1: The construction of plasmid pM09/Pro1-Hc as the heavy chain of Pro1; Table S2: The construction of plasmid pM09/Pro1-Lc as the light chain of Pro1; Table S3: The construction of plasmid pM09/Pro2-Hc as the heavy chain of Pro2; Table S4: The construction of plasmid pM09/Pro3-Hc as the heavy chain of Pro3.

## Appendix A

### Appendix A.1. The DNA Sequence of Anti-PD-L1 mAb (Atezolizumab)

Variable Domain of the Heavy Chain

GAAGTGCAGTTGGTGGAGTCTGGGGGAGGCTTGGTACAGCCTGGCGGATCCCTGAGACTCTCCTGTGCAGCATCTGGATTCACCTTTTCCGATTCTTGGATTCACTGGGTCCGGCAAGCTCCAGGGAAGGGCCTGGAGTGGGTCGCATGGATTAGTCCATATGGTGGTAGTACCTATTATGCGGACTCTGTGAAGGGCCGATTCACCATCTCCGCGGACACCAGTAAGAACACCGCTTATCTGCAAATGAACAGTCTGAGAGCTGAGGACACGGCCGTCTATTACTGTGCGCGAAGACACTGGCCAGGTGGCTTTGACTACTGGGGCCAGGGAACCCTGGTCACCGTCTCCTCT

Variable Domain of the Light Chain

GACATCCAGATGACCCAGTCTCCATCCTCACTGTCTGCATCTGTAGGAGACAGAGTCACCATCACTTGTCGGGCGAGTCAGGACGTAAGCACCGCCGTAGCATGGTATCAGCAGAAACCAGGTAAAGCCCCTAAGTTACTGATCTATTCCGCATCCTTCTTGTATAGTGGGGTCCCATCAAGGTTCAGCGGCAGTGGATCTGGGACAGATTTCACTCTCACCATCAGCAGCCTGCAGCCTGAAGATTTTGCAACTTATTACTGCCAACAGTATCTGTACCACCCGGCAACTTTTGGCCAGGGGACCAAGGTCGAGATCAAAAGG

### Appendix A.2. The DNA Sequence of Anti-CD47 mAb (B6H12)

Variable Domain of the Heavy Chain

GAAATTGTGTTGACACAGTCTCCAGCCACCCTGTCTTTGTCTCCAGGGGAAAGAGCCACCCTCTCCTGCAGGGCCAGTCAGACTATTAGCGACTACTTACACTGGTACCAACAGAAACCTGGCCAGGCTCCCAGGCTCCTCATCAAATTTGCATCCCAATCCATTTCTGGCATCCCAGCCAGGTTCAGTGGCAGTGGGTCTGGGACAGACTTCACTCTCACCATCAGCAGCCTAGAGCCTGAAGATTTTGCAGTTTATTACTGTCAGAATGGTCACGGCTTTCCTCGGACGTTCGGCCAAGGGACCAAGGTGGAAATCAAA

Variable Domain of the Light Chain

GACATCCAGATGACCCAGTCTCCATCCTCACTGTCTGCATCTGTAGGAGACAGAGTCACCATCACTTGTCGGGCGAGTCAGGACGTAAGCACCGCCGTAGCATGGTATCAGCAGAAACCAGGTAAAGCCCCTAAGTTACTGATCTATTCCGCATCCTTCTTGTATAGTGGGGTCCCATCAAGGTTCAGCGGCAGTGGATCTGGGACAGATTTCACTCTCACCATCAGCAGCCTGCAGCCTGAAGATTTTGCAACTTATTACTGCCAACAGTATCTGTACCACCCGGCAACTTTTGGCCAGGGGACCAAGGTCGAGATCAAAAGG

### Appendix A.3. The Nucleic Acid Sequence of CV1

GAGGAGGAGCTGCAGATCATTCAGCCTGACAAGTCCGTGTTGGTTGCAGCTGGAGAGACAGCCACTCTGCGCTGCACTATCACCTCTCTGTTCCCTGTGGGGCCCATCCAGTGGTTCAGAGGAGCTGGACCAGGCCGGGTTTTAATCTACAATCAACGCCAGGGCCCGTTCCCCCGGGTAACAACTGTTTCAGACACCACAAAGAGAAACAACATGGACTTTTCCATCCGCATCGGTAACATCACCCCAGCAGATGCCGGCACCTACTACTGTATCAAGTTCCGGAAAGGGAGCCCCGATGACGTGGAGTTTAAGTCTGGAGCAGGCACTGAGCTGTCTGTGCGCGCCAAACCCTCT
