# Supplementary material for: The Generation of Dual-Targeting Fusion Protein PD-L1/CD47 for the Inhibition of Triple-Negative Breast Cancer
Source: Biomedicines. 2022 Jul 30;10(8):1843. doi: 10.3390/biomedicines10081843 (PMC9405206; doi:10.3390/biomedicines10081843)
Supplement: Supplementary file 1 [file biomedicines-10-01843-s001.zip › biomedicines-1807126-supplementary.pdf]

biomedicines

MDPI

Supplementary Materials

Generation of Dual-targeting Fusion Protein PD-L1/CD47 for Inhibition of Triple-negative Breast Cancer

Yanlin Bian [1#], Tong Lin [1#], Tanja Jakos [1], Xiaodong Xiao [2,3], and Jianwei Zhu [1,2,3*]

Construction of novel dual-targeting proteins

In this study, all of the three novel proteins Pro1, Pro2 and Pro3 with two heavy and two light chains were fully assembled. A CV1 was fused to the C-terminus of the light chain in an anti-PD-L1 mAb backbone to create novel protein Pro1 in which an intra-molecular interchain disulfide bond (through cysteine mutation at VH G44C and VL Q100C) was introduced for higher molecular stability. (Figure S1-A) This heavy chain was identical to that of an IgG, and the DNA fragment Pro1-Hc was obtained through ligating 3 amplification reaction products by overlap PCR, with a full-length of 1347 bp. (Table S1) The light chain was constructed as leader-VL*-Cκ-(G4S)2-CV1, (Fig.S1-B). The fragment Pro1-Lc was prepared through ligating 4 amplification reaction products by overlap PCR, with a full-length of 1032 bp (Table S2). Both fragments mentioned above were cloned between the restriction enzyme site of Hind Ⅲ and BamH Ⅰ into a linear plasmid pM09/5D9 Hc, followed by confirmation of correct assembly by enzyme digestion and DNA sequencing (Figure S1-E).

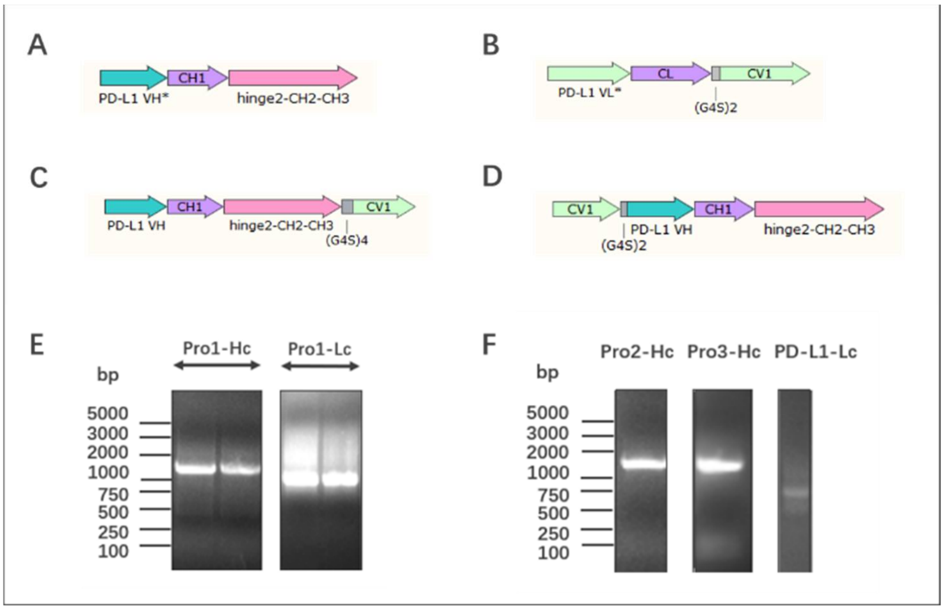

Figure S1. Molecular schematic and plasmid constructions of three dual-targetingproteins (A) Fragment Pro1-Hc (B) Fragment Pro1-Lc (C) Fragment Pro2-Hc (D) Fragment Pro3-Hc (E) DNA identification of Pro1 plasmids (F) DNA identification of Pro2 and Pro3 plasmids.

Table S1. The construction of plasmid pM09/Pro1-Hc as the heavy chain of Pro1.

Material		Code	Content	Aim
pM09/Pro1-Hc PCR system 1	Template	pM09/5D9 Hc	A targeting antibody with a IgG1 backbone heavy chain	To clone the leading sequence
	Primer F	F-p1f1	GTACAAAAAAGTTGGCACCAAGCTTACCAT GGGTTGGAGCCTCATCTTG	
	Primer R	R-p1f1	CTGCACTTCGGAGTGGACACGCGTAGCAAC	
pM09/Pro1-Hc PCR system 2	Template	pET22b/PD-L1 scfv*	A mutated anti-PD-L1 scfv with Atezolizumab variable regions(VH G44C, VL Q100C)	To clone the mutated VH targeting PD-L1
	Primer F	F-p1f2	CGTGTCCACTCCGAAGTGCAGTTGGTGGAG	

	Primer R	R-p1f2	CTTCGTCGACGCAGAGGAGACGGTGACC	
pM09/Pro1-Hc PCR system 3	Template	pM09/5D9 Hc	A targeting antibody with a IgG1 backbone heavy chain	To clone the IgG1 backbone heavy chain
	Primer F	F-p1f3	TCACCGTCTCCTCTGCGTCGACGAAGGGG	
	Primer R	FcR	TACAAGAAAGTTGAGGATCCTCACTTCCCGGGGCTCAG	

Table S2. The construction of plasmid pM09/Pro1-Lc as the light chain of Pro1.

Material		Code	Content	Aim
pM09/Pro1-Lc PCR system 1	Template	pM09/5D9 Hc	A targeting antibody with a IgG1 backbone heavy chain	To clone the leading sequence
	Primer F	F-p1f1	GTACAAAAAAGTTGGCACCAAGCTTACCATGGGTTGGAGCCTCATCTTG	
	Primer R	R-p2f1	CTGGATGTCGGAGTGGACACGCGTAG	
pM09/Pro1-Lc PCR system 2	Template	pET22b/PD-L1 scfv*	A mutated anti-PD-L1 scfv with Atezolizumab variable regions(VH G44C, VL Q100C)	To clone the mutated VL targeting PD-L1
	Primer F	F-p2f2	GCGTGTCCACTCCGACATCCAGATGACC	
	Primer R	R-p2f2	GGCCACGGTCCTTTTGATCTCGACCTTG	
pM09/Pro1-Lc PCR system 3	Template	pM09/CD3-Lc2	A targeting antibody with a IgG1 backbone light chain	To clone the IgG1 backbone Cκ
	Primer F	F-p2f3	AGATCAAAAGGACCGTGGCCGCCCCC	
	Primer R	R-p2f3	CTCCGCCACTACCGCCACCTCCGCACTCGCCCCGGTTGAAG	
pM09/Pro1-Lc PCR system 4	Template	pET22b/CV1	a CV1 monomer	To clone CV1 sequence
	Primer F	F-p2f4	GGAGGTGGCGGTAGTGGCGGAGGTGGTTCTGAGGAGGAGCTGCAGATC	
	Primer R	FcR	TACAAGAAAGTTGAGGATCCTCACTTCCCGGGGCTCAG	

Novel proteins Pro2 and Pro3 were subsequently constructed by CV1's fusion at the C-terminus or N-terminus of anti-PD-L1 IgG, where the heavy chain was VH-CH1-Fc-$(G_4S)_4$-CV1 (Pro2) or CV1-$(G_4S)_2$-VH-CH1-Fc (Pro3), (Figure S1-C and D), and both DNA fragments were obtained from 2 amplification reaction products by overlap PCR, with full-lengths reaching to 1764 bp and 1734 bp, respectively (Table.S3 and S4). Light chain used here, was the same as the plasmid prepared from stored constructs (pM09/PD-L1-Lc). All the plasmids mentioned above were cloned between the restriction enzyme sites of Hind Ⅲ and BamH Ⅰ into a linear plasmid pM09/5D9 Hc, which was certified by nucleic acid digestion and sequencing (Figure S1-F).

Table S3. The construction of plasmid pM09/Pro2-Hc as the heavy chain of Pro2.

Material		Code	Content	Aim
pM09/Pro2-Hc PCR system 1	Template	pM09/PD-L1-Hc	The heavy chain of an anti-PD-L1 antibody in a IgG1 backbone	To clone the heavy chain of anti-PD-L1 mAb
	Primer F	F-p1f1	GTACAAAAAAGTTGGCACCAAGCTTACCATGGGTTGGAGCCTCATCTTG	
	Primer R	R-p3f1	CTCCGCCGCTGCCACCACCACCACCGGATCCTCCTCCCCCGGCCCCGGGGCTCAGGCTCA	
pM09/Pro2-Hc PCR system 2	Template	pM09/Pro1-Lc	The light chain of Pro1 in a IgG1 backbone	To clone the CV1 sequence

	Primer F	F-p3f2	GGTGGCAGCGGCGGAGGAGGCTCAGGCGGC GGAGGGTCCGAGGAGGAGCTGCAGA	
	Primer R	FcR	TACAAGAAAGTTGAGGATCCTCACTTCCCGG GGCTCAG	

Table S4. The construction of plasmid pM09/Pro3-Hc as the heavy chain of Pro3.

Material		Code	Content	Aim
pM09/Pro3-Hc PCR system 1	Template	pM09/Pro1-Lc	The light chain of Pro1 in a IgG1 backbone	To clone the heavy chain of anti-PD-L1 mAb
	Primer F	F-p1f1	GTACAAAAAAGTTGGCACCAAGCTTACCAT GGGTTGGAGCCTCATCTTG	
	Primer R	R-p4f1	ACCTCCGCCACTACCGCCACCTCCAGAGGGT TTGGCGCGCACAGACAGC	
pM09/Pro3-Hc PCR system 2	Template	pM09/PD-L1-Hc	The heavy chain of an anti PD-L1 in a IgG1 backbone	To clone the heavy chain of anti-PD-L1 mAb
	Primer F	F-p4f2	GGCGGTAGTGGCGGAGGTGGTTCTGAAGTG CAGTTGGTGGAGTCTG	
	Primer R	FcR	TACAAGAAAGTTGAGGATCCTCACTTCCCGG GGCTCAG	
